# Change in Physical Performance Correlates with Decline in Quality of Life and Frailty Status in Head and Neck Cancer Patients Undergoing Radiation with and without Chemotherapy

**DOI:** 10.3390/cancers13071638

**Published:** 2021-04-01

**Authors:** Mark Farrugia, Kayleigh Erickson, Elizabeth Wendel, Mary E. Platek, Wenyan Ji, Kristopher Attwood, Sung Jun Ma, Fangyi Gu, Anurag K. Singh, Andrew D. Ray

**Affiliations:** 1 Department of Radiation Medicine, Roswell Park Comprehensive Cancer Center, Buffalo, NY 14203, USA; mark.farrugia@roswellpark.org (M.F.); Mary.Platek@RoswellPark.org (M.E.P.); SungJun.Ma@RoswellPark.org (S.J.M.); Anurag.Singh@RoswellPark.org (A.K.S.); 2 Department of Cancer Prevention and Control, Roswell Park Comprehensive Cancer Center, Buffalo, NY 14203, USA; Kayleigh.Erickson@RoswellPark.org (K.E.); Elizabeth.Wendel@RoswellPark.org (E.W.); Fangyi.Gu@RoswellPark.org (F.G.); 3 Department of Dietetics, D’Youville College, Buffalo, NY 14203, USA; 4 Department of Biostatistics, Roswell Park Comprehensive Cancer Center, Buffalo, NY 14203, USA; Wenyan.Ji@RoswellPark.org (W.J.); Kristopher.Attwood@RoswellPark.org (K.A.); 5 Department of Rehabilitation Sciences, Roswell Park Comprehensive Cancer Center, Buffalo, NY 14203, USA

**Keywords:** function, performance, gait speed, sarcopenia, cachexia

## Abstract

**Simple Summary:**

Quality of life (QoL) scores and frailty status are becoming increasingly important criterion with implications on both how patients are treated and survival in head and neck cancer (HNC). Despite this, physicians lack tools to identify patients who are at risk of suffering declines in QoL and becoming frail following treatment. Therefore, we investigated whether functional decline, as measured by a series of physical tests called the Short Physical Performance Battery (SPPB), correlated with a reduction in QoL and increased risk of frailty. In the current study, patients who experienced a decline in SPPB scores were significantly more likely to have changes in physical functioning QoL measures as well as transition to frail status following treatment. In conclusion, the SPPB may be a useful tool to identify patients who may benefit from additional rehabilitation in future studies.

**Abstract:**

Patient-reported quality of life (QoL) metrics, frailty status, and physical functioning are emerging concepts in head and neck cancer (HNC) with implications on both treatment decision-making and prognosis. The impact of treatment-related functional decline on QoL and frailty has not been well-characterized in HNC and was the focus of this investigation. Methods: Patients who underwent radiation therapy for HNC from 2018 to 2020 were evaluated as a prospective observational cohort. Functional decline, QoL, and the frailty phenotype were measured via the Short Physical Performance Battery (SPPB), European Organization for Research and Treatment of Cancer (EORTC) qlq-C30, and Fried Frailty index, respectively. Results: A total of 106 HNC patients were included, 75 of which received concurrent chemoradiation therapy (CCRT) and 31 received radiation alone, both with and without surgery. There was a decrease in SPPB overall (*p* < 0.001) from the beginning to the end of treatment in the CCRT group but not the radiation group (*p* = 0.43). Change in overall SPPB points following treatment correlated with the decline in physical QoL for both groups (*p* < 0.05) as well as transition frail status in the CCRT group (*p* < 0.001) with a trend in the radiation group (*p* = 0.08). Conclusions: Change in SPPB correlates with QoL and transition to frailty status in patients undergoing definitive CCRT for HNC with similar trends in those receiving radiation alone. Decline in SPPB could potentially be useful in identification of those who may benefit from rehabilitation in future studies.

## 1. Introduction

Head and neck cancers (HNC) of the mouth, salivary glands, pharynx and larynx represent one of the top 10 most diagnosed cancers for men in the United States (US), with an estimated 53,260 new cases and 10,750 deaths from the disease [1]. At least 60% of these patients will present with locally advanced, non-metastatic disease [2] that is commonly treated with radiation therapy or concurrent chemoradiation therapy (CCRT) [3]. Unfortunately, treatment for HNC is associated with considerable morbidity such as fatigue [4], dysphagia [5], anxiety and depression [6], shoulder dysfunction [7], malnourishment [8], dependence on caregivers [9], and a reduction in self-esteem [10] as well as quality of life (QoL) [11].

Patient-reported QoL measures are becoming increasingly important clinical objectives which characterize the patient experience of disease and treatment, as well as carry prognostic implications [12,13,14,15,16,17,18,19]. In particular, researchers have found post-treatment decline of global health status and physical functioning scores to be associated with survival in HNC [14,15,19]. Similarly, frailty or increased physical, functional, psychological, and social deficiencies associated with aging has been correlated with increased morbidity from treatment, reduced QoL, and survival in HNC [20,21,22,23]. Separately, physical functioning is an important factor regarding outcome in cancer patients [24,25] and rehabilitation interventions have been shown to be safe and effective at reducing impairment and improving QoL HNC [26]. However, the interplay between physical decline, QoL, and frailty is not well characterized in HNC. We hypothesize that treatment-related decline of physical performance, as measured by the Short Physical Performance Battery (SPPB), correlates with QoL and frailty status and could be used to identify patients who may benefit from early rehabilitation.

## 2. Methods

### 2.1. Design

A prospective cross-sectional design was used to assess the functional status of HNC patients undergoing radiation therapy or CCRT. Over the past 1.5 years, the research team has attended the same radiation outpatient clinic (one physician, A.S.) within a comprehensive cancer center one day per week in order to perform the assessments. Patients were approached prior to and immediately following their last treatment session. Because functional testing was included as part of their regularly scheduled appointment, every effort was made to keep the assessment as brief as possible (<10–15 min) and not to overburden the patient. At the end of treatment, the research staff performed a medical chart review to determine specific diagnoses, Human Papilloma Virus (HPV) status, treatment type, dose, and duration, as well as to assess body weight, height, body mass index (BMI m/kg^2^), and functional status using the KPS and ECOG performance status assigned by the treating physician. Human Subjects Institutional Review Board Approval was obtained for this study and all participants voluntarily participated (EDR-103707).

### 2.2. Participants

Inclusion criteria: ≥18 years of age with a diagnosis of stage I to IV HNC carcinoma scheduled to receive radiotherapy alone, CCRT, surgery with radiotherapy or surgery with CCRT, capable of understanding and adhering to the protocol requirements, and agree to participate. Exclusion Criteria: unable to follow directions.

### 2.3. Treatments

Radiation to 70 Gray over 7 weeks, and usually cisplatin-based chemotherapy (when given), were performed, as previously described [27,28].

### 2.4. The Short Physical Performance Battery

Participants performed the SPPB as previously described [29] prior to and immediately following their last treatment session. The following tests were performed: (1) standing balance, (2) 4 m gait speed, and (3) the 5 times sit-to-stand test (5-STS). Standing balance required the patients to maintain their (a) feet together, (b) semi-tandem, and (c) tandem for 10 s each. Gait speed test required patients to walk at a usual pace over a 4 m course and time was recorded in seconds. The 5-STS required the patient to rise from a standard chair five times as quickly as possible with their arms across their chest. Scores range from 0 to 4 (best) for each test and categorical scores were previously established for gait speed and sit to stand times [29]. Individuals who were unable to complete any functional test received a zero score. The sum of the three components comprised the final SPPB score (range 0 to 12). A score of 12 indicated the highest degree of lower extremity functioning [29]. Scoring < 10 total points is associated with increased risk for complications and comorbidities, including survival in cancer [30]. The time to compete the gait speed test and the 5-STS were also reported in seconds.

### 2.5. Health-Related Quality of Life (QoL)

Quality of life was assessed prior to and immediately following the last treatment session via the European Organization for Research and Treatment of Cancer (EORTC) Quality of Life Questionnaire C30 (QLQ-C30) and with the EORTC head and neck module (EORTC HN35) (Supplementary Document S1) [31]. The EORTC questionnaire is a well-validated instrument comprised of 30 questions divided into 5 functioning scales (physical, social, role, emotional, and cognitive functioning), 3 symptom scales (fatigue, nausea and vomiting, and pain), and 6 single items representing common cancer symptoms (dyspnea, appetite loss, insomnia, constipation, diarrhea, and financial impact of the disease), with higher scores presenting higher global QoL and function, and lower scores presenting lower symptom severity.

### 2.6. Frailty

The frailty phenotype was characterized by the following five conditions as described by Fried et al. [32]. Slowness or gait speed was determined with the use of a 15-foot walking test with pre-specified cut-off values based on gender and height [33]. Weakness was characterized by grip strength using pre-specified cut-off values based on gender and body mass index [33]. Exhaustion was characterized with an answer of 2 or 3 ((1) rarely or none of the time (<1 day), (2) some or a little of the time (1–2 days), (3) occasionally or a moderate amount of the time (3–4 days), and (4) most of the time (5–7 days)) on the following questions: “in the previous week, have you had any feelings like you couldn’t get going through the day” and “in the previous week, have you had any feelings like everything you did was an effort” [32]. Low Activity was classified as participants who answered “NO” to both of the following questions: “do you engage in low levels of physical exercise or sports aimed at health” and “do you engage in moderate levels of physical exercise or sports aimed at health” [34]. Weight loss was characterized by the unintentional loss of >10 pounds or 5% over the previous 12 months [32]. Participants who have none of these components were considered robust, those with difficulties in one or two of the components were considered pre-frail, and those with greater than three were considered frail.

### 2.7. Statistical Analysis

Characteristics and analyses were compared between the two treatment groups: CCRT and radiotherapy (RT). Regarding comparisons for continuous, ordinal, and categorical variables, the Student’s *t*-test, Mann–Whitney U test, and χ^2^ or Fisher’s exact test were used, respectively. For pre- and post-treatment comparisons within the CCRT and RT groups, Wilcoxon test was used. General linear models were used to compare relationships between SPPB, QoL, and frailty parameters. Patients who were unable to complete a given test were given the maximum/minimum (based on which values correspond to poorer outcomes) values observed within the sample. Statistical analysis was completed by SPSS Statistics for Windows, version 25 (SPSS Inc., Chicago, IL, USA).

## 3. Results

### 3.1. Patient Demographics

A total of 106 HNC patients were included, of which *n* = 75 received CCRT and *n* = 31 received radiation alone, both with and without surgery (Table 1). The combined groups were primarily Caucasian (~88%), male (~75%), and half were HPV+. Prior to treatment, two CCRT patients could not complete any functional assessment and seven additional patients were unable to complete the 5-STS. Following treatment, 10 CCRT patients were unable to complete any of the functional assessments and two were unable to perform the 5-STS. At baseline, seven radiation patients did not complete the sit-to-stand test and two could not complete the balance tests. Post-treatment, five radiation patients were unable to perform all the SPPB tests, whereas four could not perform the sit-to-stand test.

Overall, there were differences between groups prior to beginning their cancer treatment (Table 1): the radiation group was older (*p* < 0.001), more functionally compromised as measured by the KPS (*p* < 0.001) and ECOG (*p* < 0.01), they were shorter (*p* < 0.05) and trended towards a higher BMI (*p* = 0.07), they were more likely to undergo surgery prior to treatment (*p* < 0.05), they were also less likely to have a cancer of the pharynx (*p* < 0.01), and their treatment occurred over a shorter number of days (43.0 ± 3.4 vs. 45.7 ± 4.3, *p* < 0.01), at a lower overall dose (66.8 ± 3.7 vs. 68.9 ± 3.9, *p* < 0.05), and with a smaller number of fractions (33.1 ± 2.3 vs. 34.6 ± 1.1, *p* < 0.001), compared to the CCRT group.

### 3.2. Pre- and Post-Treatment Comparisons

Within the CCRT group, body weight (87.6 ± 20.7 kg vs. 80.7 ± 18.5 kg, *p* < 0.001) and body mass index (BMI) (28.7 ± 6.1 vs. 26.6 ± 5.4, *p* < 0.001) both decreased from the beginning to the end of treatment, respectively. The CCRT group demonstrated a decrease in KPS (*p* < 0.001) and ECOG status (*p* < 0.001) from the beginning to the end of treatment. Similar treatment-related changes were observed in the radiation only group, including body weight (84.7 ± 23.2 kg vs. 80.0 ± 21.2 kg, *p* < 0.001), BMI (29.5 ± 7.4 m/kg^2^ vs. 27.5 ± 6.5 m/kg^2^, *p* < 0.001), KPS (*p* < 0.042), and ECOG status (*p* < 0.049).

### 3.3. Short Physical Performance Battery

**Figure 1:**
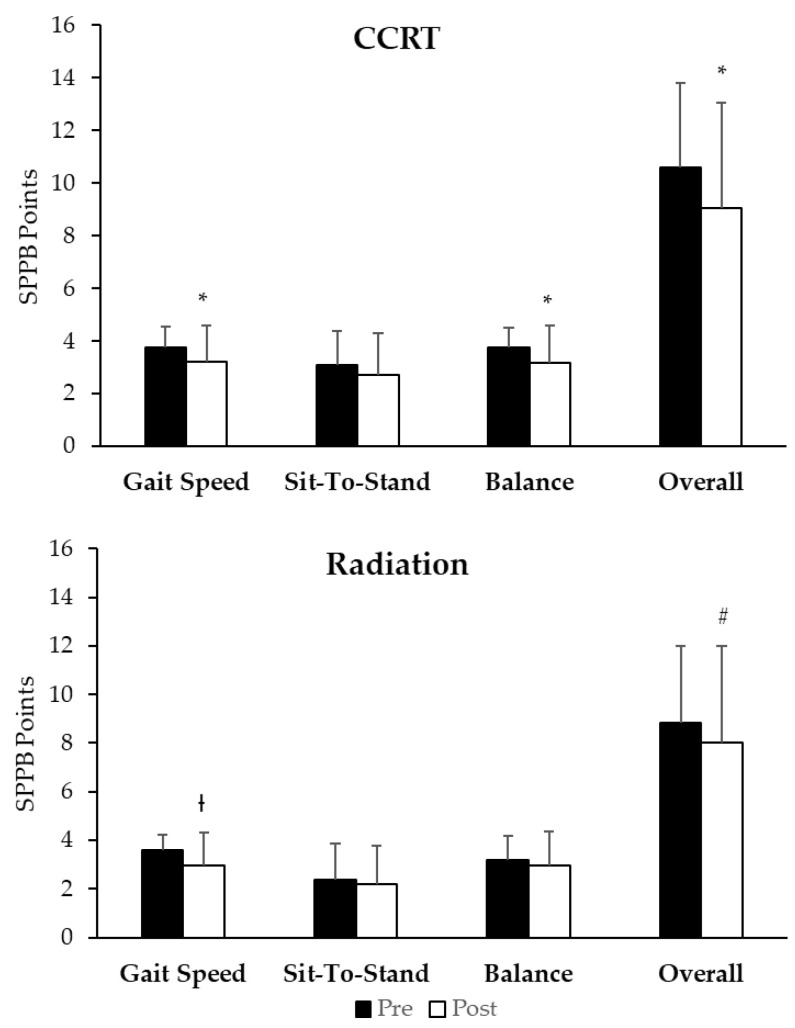
Short Physical Performance Battery (SPPB) scores in each category pre- (black) and post-treatment (white) for patients who received CCRT or radiation alone. Statistical significance indicated by * (*p* < 0.001), † (*p* = 0.009), and # (*p* = 0.01).

Although, the CCRT group had a tendency to outperform the radiation group in all categories of the SPPB prior to and immediately following cancer treatment (Figure 1), the overall SPPB and each subcategory (gait, sit-to-stand, and balance) were not significantly different between the two groups before or immediately following treatment. In the CCRT group, gait speed (*p* < 0.001), sit-to-stand (*p* = 0.083), balance (*p* < 0.001), and overall (*p* < 0.001) points declined following treatment. Whereas only gait speed declined (*p* = 0.009) and overall (*p* = 0.01) points in the radiation group following the last treatment. When examining the delta or change in points from the beginning to the end of treatment, there was no difference between the CCRT and radiation groups for gait speed (*p* = 0.628), sit-to-stand (*p* = 0.613), balance (*p* = 0.138), and overall points (*p* = 0.433), even with the radiation group starting out with lower scores.

### 3.4. Quality of Life

Mean differences in health-related QoL at baseline and again following treatment can be seen in Table 2 for both groups. Within the CCRT group, global QoL, along with four out of the five physical subscales (physical, role, cognitive, and social), were all lower by the end of treatment, with the lone exception for emotional QoL. Within the radiation group, global QoL, along with three of the five physical subscales (physical, role, and social), declined by the end of treatment, while emotional and cognitive QoL did not change with treatment. Finally, the differences in pre- to post-scores for all QoL categories were not different between groups. The only difference in QoL between groups prior to and immediately following treatment was a higher score on the dyspnea symptom scale (more dyspnea) in the radiation group at baseline.

Quality of life was measured with the EORTC QLQ-30 before the first (Pre) and following the last (Post) treatment session. Patients received radiation (*n* = 31) or concurrent chemoradiation (CCRT, *n* = 76). Values represent means ± standard deviation (SD). Comparisons were done from pre- to post-treatment within each group, between groups pre-treatment, between groups post-treatment, and the change in QoL from pre- to post-treatment was compared between the two groups.

### 3.5. SPPB and QoL

**Figure 2:**
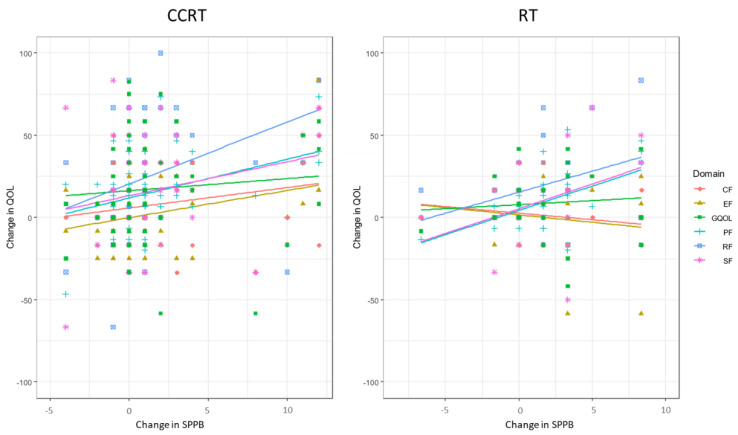
Correlation between the change in physical functional and global QOL and SPPB for patients who received CCRT or radiation alone (RT). CF = cognitive function, EF = emotional function, GQOL = global quality of life, PF = physical function, RF = role physical function, and SF = social function.

The relationship between functional performance, as measured with the SPPB, with self-reported QoL is presented in Table 3 for the CCRT group. Within the CCRT group, performance on the SPPB correlated with the following QoL categories prior to treatment: global, physical, role, social, and fatigue. Post-treatment, the SPPB correlated with global, physical, role, emotional, cognitive, social, fatigue, pain, dyspnea, insomnia, as well as financial QoL. Finally, the decline in performance on the SPPB correlated with a worsening of self-reported physical, role, and social QoL from the beginning to the end of treatment (Table 3, Figure 2).

The relationship between the SPPB with self-report QoL is presented in Table 4 for the radiation group. Prior to cancer therapy, the SPPB did not correlate with any self-reported measures of QoL. In contrast, performance on the SPPB correlated with self-reported physical, role, social, fatigue, pain, and diarrhea QoL at the completion of treatment. Finally, the decline in SPPB performance from the beginning to the end of treatment only correlated with a decline in physical QoL in the radiation group (Table 4, Figure 2).

### 3.6. Frailty

Distributions of robust, pre-frail, and frail patients in the CCRT and radiation groups are shown in Table 5. Patients undergoing CCRT were less frail prior to therapy (*p* = 0.021) and after treatment (*p* = 0.036). There was a significant increase of the frail phenotype following treatment within both the CCRT (*p* < 0.001) and RT (*p* < 0.001) groups. The relationship between frailty and functional status is presented in Table 6. Pre-treatment SPPB measures significantly correlated with frailty status for the CCRT group but not radiation. In contrast, post-treatment SPPB metrics correlated with frailty status in both groups. To investigate the relationship between change in SPPB scores and change in frailty status, we evaluated patients who transitioned from robust/pre-frail to frail following treatment. Within the CCRT and radiation groups respectively, 23 patients (30.7%) and 15 patients (48.4%) transitioned to frail after treatment. SPPB decline was significantly associated with transition to frail status in the CCRT group (Beta 0.048 (Standard Error (SE) 0.016), *p* = 0.004) but not the radiation group (Beta 0.073 (SE 0.041), *p* = 0.084). Additionally, transition to frail correlated with QoL and symptom scores (Supplementary Table S1).

## 4. Discussion

We incorporated the Short Physical Battery of Tests to characterize the change in performance commonly seen in HNC patients receiving radiation with or without chemotherapy. Those undergoing CCRT were distinct from the radiation group, as they were significantly younger, had better KPS and ECOG metrics, and had different aspects of treatment. Nevertheless, following treatment, both groups experienced decline in performance status, weight, gait speed, QoL metrics, as well as increased frailty. Change in SPPB was significantly correlated with physical functional domain scores among both groups and transition to frailty in the CCRT group, with a trend observed in the radiation group.

The SPPB has emerged as a promising tool in cancer and non-cancer populations [29]. In gynecological malignancies, the SPPB was able to predict survival, as well as a one-point reduction in the SPPB was associated with a 65% chance of functional decline [25]. In contrast, a one-point increase in the SPPB is associated with a 12% reduction in mortality among cancer survivors [24], as well as a 28% decrease in adverse events, hospitalizations, and delays in lung cancer treatment [35]. To our knowledge, only one other study has incorporated the SPPB in patients with HNC [36]. In comparison, total baseline SPPB points in the current CCRT group (10.6 ± 3.2), but not the radiation group (8.9 ± 3.1), were similar to those reported by Saerol et al. in HNC patients (10.4 ± 2.9) [36]. We suggest a reason for the reduced performance in the radiation only group may reflect the different disease sites as well as the older age of this group, which is commonly associated with an increased risk of sarcopenia even prior to treatment [37]. In support, concurrent chemotherapy is not recommended for definitive treatment of HNC in patients older than 70 years of age [38]. The lower baseline and post-treatment scores seen in the radiation group are also below a pre-established threshold (<10/12 points), which defines an increased risk for malnutrition, morbidity, and mortality, even prior to the onset of treatment [30].

Generally, QoL declines during and immediately following treatment: these may normalize as early as 3 months following treatment [39] or take 12 months or longer to improve once treatment has ceased [40,41,42]. Our self-reported QoL scores for both the CCRT and RT groups are comparable to values obtained in similar HNC studies [43,44,45]. The lower scores in the radiation only group are consistent with older age and more functional compromise, as demonstrated by their poorer ECOG scores. Furthermore, being older and more functionally compromised is generally associated with a sedentary lifestyle and/or a reduced amount of physical activity, which directly correlates with QoL in HNC [46].

Several QoL domains have been associated with survival in HNC [12,13,14,15,16,17,18,47,48,49]. Typically, groups have shown utility as a baseline score, however others have investigated change in QoL metrics and outcome [15,19]. Meyer et al. demonstrated that the pre-treatment physical functioning score and relative change 6 months following treatment was independently associated with survival in HNC [14]. Furthermore, decline in global health status following treatment is associated with increased mortality in HNC [15,19]. Previous work by our group utilized principal component analysis to generate a composite score for recovery across several functional domains (physical, role, emotional, cognitive, global health status) at three months, observing poorer recovery to associate with increased mortality (Manuscript under review).

There were several differences between the correlations observed within the CCRT and RT groups, such as lack of correlation between pre-treatment SPPB with physical functioning scores as well as change in SPPB only correlating with the change of a single functional domain in the RT group. Radiation alone is a therapeutic option for early-stage disease in certain HNC subsites, in select post-operative patients, and in elderly patients, as addition of concurrent chemotherapy to radiotherapy did not improve survival within HNC patients 70 years or older [38]. As such, the RT group was significantly older and more functionally compromised as compared to the CCRT. Additionally, the difference in sample size between the two populations may have contributed to these findings. In Figure 2, the trend lines for physical, role, and social functioning domains were similar between the two groups, yet only change in physical functioning was significantly associated in both groups. It is possible that with additional patients, similar findings would have been seen the RT group.

In the current study, change in SPPB was associated with physical function scores in the CCRT group with a trend in the radiation group. Moreover, delta SPPB correlated with transition to frail status following treatment. Frailty is an emerging concept in oncology, with over half of elderly cancer patients considered pre-frail or frail demonstrating increased risk of chemotherapy intolerance, post-treatment complications, and mortality [50,51]. Many studies have shown that frailty status was associated with increased post-operative complications in HNC [21,22,52,53]. In both surgical and radiation therapy HNC patients, frailty status correlates with diminished QoL measures across several functional domains and those with pre-treatment deficits were at an increased risk of swallowing and respiratory dysfunction as well as a further QoL decline post-treatment [20,54,55]. Low-intensity walking programs have been shown to improve frailty parameters in elderly patients [56]. Therefore, with the combined association of change in SPPB with physical function scores and frailty, rehabilitation programs are an attractive strategy to mitigate the clinical consequences of post-treatment decline. Rehabilitation programs implemented during HNC treatment can preserve muscle mass as well as maintain or improve physical QoL [57]. Focusing on difference of pre- and post-treatment measures adjusts for baseline deficits, thereby isolating patients who were particularly impacted by treatment. This patient population may be better suited to benefit from interventions such as rehabilitation. Thus, addressing deficits early may improve short- and long-term QoL as well as survival outcomes by preserving muscle mass and quality.

Notably, we correlated function with QoL over time and did not examine which measure will more accurately predict a decline in functional performance (SPPB, EORTC, frailty). Our initial evidence suggests that EORTC may represent a more sensitive and less burdensome approach towards quantifying a decline in function for both cohorts. Future studies are needed to determine the best approach to identify patients at risk for treatment-related functional impairments who may benefit from rehabilitation or pre-habilitation.

An important component in HNC treatment, particularly within the elderly and frail population, is comorbidity burden. Comorbidity burden, as measured by indices such as the Adult Comorbidity Evaluation 27 (ACE 27), Charlson Index (CI), and Cumulative Illness Rating Scale, has been significantly associated with mortality and patient QoL [58,59]. While performance status was recorded in the current study, several reports have shown that it is not a replacement for comorbidity burden [58]. This is supported by the observation that comorbidity burden is an independent prognostic factor in HNC even when adjusting for performance status [60,61,62]. Future studies should consider investigating how patient morbidity impacts physical performance during treatment.

The SPPB represents a series of tests that require additional training and time to administer, making it difficult to integrate into a busy clinical practice. Although the SPPB was successful, other strategies (i.e., gait speed and/or a 30 s sit-to-stand test) may represent a more realistic approach to quantify physical performance in a busy outpatient clinic.

## 5. Conclusions

In summary, the SPPB was associated with pre-treatment and post-treatment QoL in both groups, with a significant relationship between change in SPPB and the physical functioning domain in the CCRT group. Change in SPPB was significantly associated with transition to frail status following treatment in the CCRT group with a trend observed in the radiation group.

## Figures and Tables

**Table 1:** Patient demographics.

	Overall	CCRT	Radiation	*p*-Value
# (%)	106 (100)	75 (70.8)	31 (29.2)	
Age (Years)	64.0 ± 10.5	61.5 ± 9.0	69.3 ± 11.5	<0.001
Male (%)	74.5	76.4	70.6	0.33
ECOG				<0.01
0	65.1	74.7	41.9	
1	34.9	25.3	58.1	
KPS (%)				<0.001
100	21.7	25.3	12.9	
90	44.3	50.7	29	
80	25.5	18.7	41.9	
70	8.5	5.3	16.1	
Caucasian (%)	87.7	84.7	94.1	0.76
Height (cm)	173.0 ± 9.6	174.5 ± 9.8	170.1 ± 8.4	<0.05
Weight (Kg)	86.7 ± 21.5	87.6 ± 20.7	84.7 ± 24.0	0.51
BMI (kg/m^2^)	29.0 ± 6.6	28.7 ± 6.1	29.5 ± 7.4	0.07
HPV+ (%)	51.9	59.7	35.3	0.09
Surgery (%)	36.8	26.4	58.8	<0.05
Smoking (%)				0.39
Former	56.6	56.9	55.9	
Never	35.8	37.5	32.4	
Current	6.6	5.6	8.8	
Unknown	0.9	0	2.9	
Site (%)				<0.01
Pharynx	61.3	72.2	38.2	
Larynx	17.9	13.9	26.5	
Lip/Oral Cavity	11.3	5.6	23.5	
Other	9.4	8.4	11.7	
Stage (%)				0.1
I	23.1	26.7	13.8	
II	21.1	16	34.4	
III	26	24	31	
IV	29.8	33.3	20.7	
Treatment Days	44.9 ± 4.2	45.7 ± 4.3	43.0 ± 3.4	<0.01
Dose	68.3 ± 3.9	68.9 ± 3.9	66.8 ± 3.7	<0.05
Fraction	34 ± 1.7	34.6 ± 1.1	33.1 ± 2.3	<0.001

Values represent means ± standard deviation or a percentage. CCRT = Concurrent Chemoradiation Therapy, HPV+ = Human Papilloma Virus positive; BMI = body mass index; KPS = Karnosky Performance Status; ECOG = Eastern Cooperative Oncology Group.

**Table 2:** Quality of life.

	CCRT			Radiation			Pre	Post	Change
	Pre	Post	*p*-Value	Pre	Post	*p*-Value	CCRT vs. Radiation	CCRT vs. Radiation	CCRT vs. Radiation
**Functional**									
Global	73.0 ± 24.3	56.5 ± 23.8	<0.001	67.5 ± 20.8	57.5 ± 27.5	0.023	0.119	0.521	0.381
Physical	90.4 ± 18.7	76.1 ± 22.3	<0.001	80.0 ± 19.5	69.5 ± 28.2	0.019	0.077	0.262	0.488
Role	85.2 ± 27.1	60.2 ± 30.8	<0.001	76.4 ± 31.9	59.1 ± 34.7	0.001	0.249	0.579	0.583
Emotional	74.7 ± 22.1	73.5 ± 24.0	0.628	78.1 ± 17.7	76.3 ± 18.7	0.301	0.908	0.725	0.793
Cognitive	85.9 ± 18.2	79.4 ± 25.5	0.005	88.9 ± 14.7	83.9 ± 20.0	0.458	0.941	0.629	0.536
Social	83.1 ± 23.0	68.1 ± 27.3	<0.001	81.1 ± 23.1	70.4 ± 30.9	0.050	0.851	0.791	0.689
**Symptoms**									
Fatigue	76.7 ± 23.4	49.6 ± 26.6	<0.001	69.3 ± 19.7	53.1 ± 28.7	0.009	0.184	0.682	0.099
Nausea	4.2 ± 14.3	17.9 ± 23.6	<0.001	5.0 ± 10.9	14.0 ± 23.6	0.021	0.417	0.269	0.139
Pain	24.9 ± 29.3	44.1 ± 25.4	<0.001	22.7 ± 24.6	45.2 ± 30.0	<0.001	0.935	0.591	0.655
Dyspnea	9.4 ± 17.1	17.9 ± 23.6	0.004	17.2 ± 25.6	17.2 ± 28.4	1.00	0.045	0.556	0.175
Insomnia	34.7 ± 33.1	39.2 ± 29.9	0.094	22.2 ± 26.7	32.3 ± 27.9	0.135	0.289	0.729	0.509
Appetite Loss	14.1 ± 23.7	48.5 ± 34.3	<0.001	22.2 ± 28.1	51.6 ± 37.4	0.002	0.230	0.289	0.919
Constipation	9.9 ± 21.4	26.0 ± 27.5	<0.001	16.7 ± 17.0	23.7 ± 37.4	0.205	0.089	0.554	0.318
Diarrhea	5.6 ± 15.9	13.2 ± 20.1	0.003	8.9 ± 17.4	15.1 ± 22.0	0.356	0.357	0.380	0.983
Financial	16.0 ± 27.0	16.7 ± 26.1	0.825	25.6 ± 27.2	19.4 ± 29.5	0.107	0.064	0.517	0.183

**Table 3:** Correlation between the SPPB and quality of life (QoL) for the CCRT group.

	Pre		Post			Change in QoL	
	Beta (SE)	*p*-Value	Beta (SE)	*p*-Value	Pre–Post	Beta (SE)	*p*-Value
Global	0.012 (0.004)	0.010	0.022 (0.004)	<0.001	0.095	0.001 (0.005)	0.786
**Functional**							
Physical	0.028 (0.005)	<0.001	0.032 (0.004)	<0.001	0.584	0.031 (0.007)	<0.001
Role	0.014 (0.004)	<0.001	0.019 (0.003)	<0.001	0.284	0.010 (0.004)	0.017
Emotional	0.007 (0.005)	0.178	0.019 (0.005)	<0.001	0.057	0.008 (0.007)	0.241
Cognitive	0.010 (0.006)	0.103	0.017 (0.004)	<0.001	0.305	0.011 (0.007)	0.126
Social	0.009 (0.005)	0.062	0.018 (0.004)	<0.001	0.124	0.010 (0.005)	0.038
**Symptoms**							
Fatigue	0.013 (0.004)	0.006	0.024 (0.004)	<0.001	0.058	0.010 (0.006)	0.080
Nausea	0.004 (0.008)	0.617	−0.005 (0.004)	0.309	0.345	0.001 (0.005)	0.870
Pain	−0.006 (0.004)	0.132	−0.011 (0.005)	0.012	0.331	0.001 (0.005)	0.769
Dyspnea	−0.005 (0.006)	0.397	−0.017 (0.005)	<0.001	0.113	−0.013 (0.007)	0.067
Insomnia	−0.002 (0.003)	0.532	−0.010 (0.004)	0.006	0.089	0.001 (0.004)	0.821
Appetite Loss	−0.003 (0.005)	0.590	−0.006 (0.003)	0.094	0.626	0.003 (0.004)	0.456
Constipation	0.000 (0.005)	0.948	0.001 (0.004)	0.755	0.881	0.002 (0.006)	0.699
Diarrhea	−0.013 (0.007)	0.087	0.004 (0.006)	0.526	0.082	0.005 (0.006)	0.457
Financial	−0.001 (0.004)	0.817	−0.013 (0.004)	0.003	0.038	−0.002 (0.006)	0.760

**Table 4:** Correlation between the SPPB and quality of life for the radiation group.

	Pre		Post			Change in QoL	
	Beta (SE)	*p*-Value	Beta (SE)	*p*-Value	Pre–Post	Beta (SE)	*p*-Value
Global	−0.002 (0.009)	0.824	0.011 (0.007)	0.117	0.144	0.008 (0.010)	0.438
**Functional**							
Physical	0.005 (0.007)	0.503	0.031 (0.005)	<0.001	0.001	0.020 (0.008)	0.022
Role	0.008 (0.005)	0.089	0.026 (0.004)	<0.001	0.003	0.007 (0.006)	0.295
Emotional	0.004 (0.011)	0.689	−0.000 (0.010)	0.996	0.712	0.002 (0.011)	0.857
Cognitive	0.004 (0.012)	0.748	0.017 (0.010)	0.074	0.307	0.004 (0.012)	0.760
Social	0.012 (0.007)	0.122	0.018 (0.006)	0.001	0.470	0.012 (0.006)	0.070
**Symptoms**							
Fatigue	0.003 (0.009)	0.770	0.016 (0.006)	0.006	0.171	0.010 (0.006)	0.142
Nausea	−0.019 (0.020)	0.348	−0.010 (0.008)	0.219	0.614	−0.008 (0.009)	0.369
Pain	−0.004 (0.007)	0.550	−0.019 (0.006)	0.001	0.058	−0.006 (0.007)	0.455
Dyspnea	0.003 (0.007)	0.629	0.002 (0.006)	0.709	0.891	−0.000 (0.006)	0.938
Insomnia	−0.004 (0.007)	0.570	0.008 (0.006)	0.219	0.175	−0.004 (0.005)	0.511
Appetite Loss	−0.002 (0.006)	0.742	−0.008 (0.005)	0.083	0.395	−0.006 (0.004)	0.156
Constipation	−0.003 (0.010)	0.794	−0.004 (0.006)	0.474	0.906	−0.005 (0.005)	0.319
Diarrhea	0.008 (0.009)	0.356	−0.022 (0.007)	0.003	0.018	−0.003 (0.007)	0.654
Financial	−0.006 (0.007)	0.421	−0.007 (0.006)	0.231	0.840	0.002 (0.006)	0.765

**Table 5:** Frailty distribution.

	Treatment	Category	Pre-Treatment	Post-Treatment
Frailty	CCRT	Robust	32 (42.7%)	3 (4.1%)
		Pre-frail	35 (46.7%)	40 (54.8%)
		Frail	8 (10.7%)	30 (41.1%)
	RT	Robust	6 (19.4%)	1 (3.2%)
		Pre-frail	19 (61.3%)	10 (32.3%)
		Frail	6 (19.4%)	20 (64.5%)

**Table 6:** Correlation between SBBP and frailty status.

		Pre-Treatment SPPB (Beta, (SE))	*p*-Value	Post-Treatment SPPB (Beta, (SE))	*p*-Value	Delta SPPB and Transition to Frail (Beta, (SE))	*p*-Value
**Frailty**	CCRT	−0.151 (0.28)	<0.001	−0.088 (0.015)	<0.001	0.048 (0.016)	0.004
	RT	−0.043 (0.036)	0.246	−0.067 (0.022)	0.004	0.073 (0.041)	0.084

## Data Availability

Farrugia, Ray, and Singh had full access to all the data in the study and take responsibility for the integrity of the data and the accuracy of the data analysis. The data underlying this article cannot be shared publicly for the privacy of individuals that participated in the study. The data are available from the corresponding author upon reasonable request.

## Supplementary Materials

The following are available online at https://www.mdpi.com/article/10.3390/cancers13071638/s1, Table S1: Frailty and Quality of life, Document S1: EORTC-QLQ-C30.
