# Supplementary material for: Change in Physical Performance Correlates with Decline in Quality of Life and Frailty Status in Head and Neck Cancer Patients Undergoing Radiation with and without Chemotherapy
Source: Cancers (Basel). 2021 Apr 1;13(7):1638. doi: 10.3390/cancers13071638 (PMC8037640; doi:10.3390/cancers13071638)
Supplement: Supplementary file 1 [file cancers-13-01638-s001.zip › cancers-1116350-supplementary-xml/Supplementary table 1. frailty and qol.pdf]

Table S1. Frailty and Quality of life.

		QoL frail (Beta, SE)	*p*-value
**Global**		−0.004 (0.002)	0.079
**Functional**	Physical	−0.007 (0.003)	0.007
	Role	−0.004 (0.002)	0.029
	Emotion	−0.010 (0.002)	<0.001
	Cognitive	−0.008 (0.002)	<0.001
	Social	−0.005 (0.002)	0.023
**Symptoms**			
	Fatigue	−0.008 (0.002)	<0.001
	Nausea and vomiting	0.007 (0.002)	0.002
	Pain	0.003 (0.002)	0.239
	Dyspnea	0.007 (0.003)	0.009
	Insomnia	0.002 (0.002)	0.443
	Appetite	0.002 (0.002)	0.173
	Constipation	0.003 (0.002)	0.147
	Diarrhea	0.005 (0.003)	0.099
